# Cultural Dimensions, Unique Challenges, and Interventions for Dementia Care in the Asian Context

**DOI:** 10.1093/geroni/igaa057.1759

**Published:** 2020-12-16

**Authors:** Kyong Hee Chee, Farida Ejaz

## Abstract

Asians in and outside of Asia are facing a rapidly-growing need for dementia care in familial, institutional, or community settings. This multidisciplinary symposium addresses issues of formal and informal dementia care in the Asian cultural context to suggest novel, culturally-appropriate interventions for education and practice. Presenters in this symposium will specifically speak to cultural dimensions, challenges, and approaches involved with caring for persons living with dementia (PLWD) in Singapore and South Korea, as well as their Japanese/Japanese-American counterparts. Malhotra and colleagues will present their qualitative study from Singapore on 26 familial care partners’ preference for life-extending interventions for persons with severe dementia, such as intravenous antibiotics, tube feeding, and cardiopulmonary resuscitation. Lee and Chee will then examine how the occupational identities of 303 long-term care workers are associated with their practice of human rights for PLWD in South Korea. Next, Yen and Mayen-Cho will explain a video project that they developed at Alzheimer’s Los Angeles to reach out to the Japanese American community – they conducted and filmed in-depth interviews with 7 Japanese/Japanese American family care partners of PLWD. Finally, Park will examine the emergent themes in the narratives of these Japanese/Japanese American interviewees. She will also demonstrate the relevance of the life-course perspective in developing a template for designing similar interventions to serve other ethnic communities. As a discussant, Ejaz will highlight versatility in interventions for dementia care among Asians and Asian Americans. She will also discuss broader implications of the findings within and beyond the Asian context. Aging Among Asians Interest Group Sponsored Symposium.

